# The Impact of PRAC EMA/AIFA Recommendations on the Prescriptions of JAKi and b-DMARDs: Preliminary Results of the Survey from 21 Rheumatological Italian Centers Affiliated with CReI

**DOI:** 10.3390/jpm16020107

**Published:** 2026-02-10

**Authors:** Emanuele Antonio Maria Cassarà, Daniela Marotto, Crescenzio Bentivenga, Luis-Severino Martin Martin, Gianpiero Baldi, Norma Carrozzo, Raffaele Zicolella, Riccardo Terenzi, Andrea Delle Sedie, Maurizio Benucci

**Affiliations:** 1 Rheumatology Unit, Hospital S. Giovanni di Dio, Via di Torregalli n° 3, 50143 Florence, Italy; riccardo.terenzi@uslcentro.toscana.it (R.T.); maurizio.benucci@uslcentro.toscana.it (M.B.); 2 Rheumatology Service, ASL Gallura, 07026 Olbia, Italy; daniela.marotto@tiscali.it; 3 Internal Medicine Unit, S. Orsola Malpighi Polyclinic, 40138 Bologna, Italy; crescenzio.bentivenga@aosp.bo.it; 4 Internal Medicine Hospital, Velletri, 00045 Rome, Italy; severino.martin@gmail.com; 5 Rheumatology Clinic, ASL 4, 00053 Rome, Italy; gianpierobaldi@libero.it; 6 Rheumatology Clinic, 72100 Brindisi, Italy; norma.carrozzo@gmail.com; 7 Rheumatology Unit, Santo Spirito Hospital, 65124 Pescara, Italy; raffaele.zicolella@ausl.pe.it; 8 Rheumatology Unit, Department of Clinical and Experimental Medicine, University of Pisa, 56124 Pisa, Italy; a.dellesedie@ao-pisa.toscana.it

**Keywords:** Jak-inhibitors, cardiovascular disease, PRAC-EMA-AIFA recommendation, Italian survey

## Abstract

**Objective:** To evaluate the impact of recommendations issued by the Pharmacovigilance Risk Assessment Committee (PRAC) and endorsed by the European Medicines Agency (EMA) and the Italian Medicines Agency (AIFA) on rheumatologists’ prescribing patterns of Janus kinase inhibitors (JAK inhibitors) and biologic disease-modifying antirheumatic drugs (bDMARDs) in patients with rheumatoid arthritis (RA), within a personalized, risk-adapted care framework. **Methods:** A brief survey was conducted across 21 Italian rheumatology centers. This retrospective multicenter study included 4421 RA patients assessed before PRAC recommendations (1 January 2022–1 January 2023) and 4376 patients evaluated afterward (2 January 2023–1 January 2024). Prescribing behaviors, cardiovascular risk management, and clinical outcomes were compared between cohorts. **Results:** Following PRAC recommendations, a more individualized cardiovascular risk management strategy was observed, with increased use of targeted treatments for hypercholesterolemia, hypertension, and diabetes. The post-PRAC cohort showed a significant reduction in myocardial infarction incidence (0.90% vs. 0.47%; *p* = 0.02) and increased statin use (8.25% vs. 11.1%; *p* = 0.05). No increase in cardiovascular risk was observed among JAK inhibitor users. Notably, upadacitinib utilization remained stable despite regulatory restrictions. **Conclusions:** PRAC recommendations promoted safer prescribing practices and improved cardiovascular risk stratification in RA. These findings support a shift toward precision medicine, integrating real-world evidence with advanced diagnostic and decision-support tools, including future artificial intelligence-based approaches, to optimize personalized therapeutic strategies in autoimmune diseases.

## 1. Introduction

Rheumatoid arthritis (RA) is a chronic autoimmune disease characterized by synovial inflammation and systemic immune dysregulation, driven by complex interactions between immune cells and soluble mediators, including cytokines [1]. Clinically, RA manifests with both articular and extra-articular involvement, contributing to significant morbidity and reduced quality of life [1]. Contemporary therapeutic strategies increasingly aim to define disease pathophenotypes and selectively target key inflammatory pathways, reflecting a paradigm shift toward precision medicine in autoimmune and rheumatic diseases.

In routine clinical practice, composite disease activity indices—such as the Disease Activity Score in 28 joints (DAS28), the Clinical Disease Activity Index (CDAI), and the Simplified Disease Activity Index (SDAI)—are widely used to objectively assess disease activity and monitor treatment response over time [2]. While these tools remain central to clinical decision making, emerging molecular profiling approaches, real-world data integration, and advanced analytics are progressively refining diagnostic accuracy and therapeutic stratification, enabling more personalized and effective care.

The Janus kinase (JAK)–signal transducer and activator of transcription (STAT) signaling pathway represents a pivotal mechanism underlying cytokine-mediated immune activation in RA [3]. JAK-dependent signaling regulates immune cell development, differentiation, and effector functions, playing a critical role in sustaining chronic inflammation [4]. Janus kinase inhibitors (JAKis) are orally administered small-molecule agents that disrupt intracellular cytokine signaling by selectively inhibiting JAK activity. Through this mechanism, JAKis modulate immune activation across multiple cellular compartments relevant to RA pathogenesis [5] and have therefore become an important therapeutic option in RA management. Currently, baricitinib (4 or 2 mg once daily), tofacitinib (5 mg twice daily), upadacitinib (15 mg once daily), and filgotinib (200 or 100 mg once daily) are approved for the treatment of RA by the European Medicines Agency, with all except filgotinib also approved by the U.S. Food and Drug Administration [6]. Since 2019, JAK inhibitors have been recommended as second-line therapy for RA, demonstrating efficacy and overall safety profiles comparable to those of biologic disease-modifying antirheumatic drugs (bDMARDs).

However, the publication of the ORAL Surveillance study in January 2022 and subsequent 2023 EULAR recommendations raised concerns regarding cardiovascular and malignancy risks associated with JAK inhibitor therapy in selected patient populations [7,8]. Specifically, clinicians are advised to carefully assess age > 65 years, smoking status, prior thromboembolic events, and history of malignancy before initiating JAK inhibitor treatment. Notably, long-term extension studies and real-world registry data have not consistently replicated the safety signals observed in ORAL Surveillance [9,10], and multiple analyses have confirmed comparable efficacy and safety profiles across JAK inhibitors, despite differences in kinase selectivity and cellular targeting [11,12].

On 11 November 2022, the Pharmacovigilance Risk Assessment Committee (PRAC) recommended that JAK inhibitors should be used with caution and reserved for patients at increased risk of major adverse cardiovascular events (MACE), malignancy, or venous thromboembolism when no suitable therapeutic alternatives are available [13,14] (Table 1). These regulatory measures have further emphasized the need for refined risk stratification, individualized treatment decisions, and integration of traditional clinical assessment with emerging data-driven tools.

In this evolving landscape, multidisciplinary approaches that combine clinical expertise, pharmacovigilance data, real-world evidence, and advanced analytical methodologies—including future applications of artificial intelligence—hold promise for optimizing therapeutic selection and improving outcomes in RA. In this context, we conducted a survey across 21 Italian rheumatology centers to evaluate the impact of PRAC recommendations on JAK inhibitor (ts-DMARDs) and bDMARD prescribing patterns in patients with RA.

## 2. Materials and Methods

This multicenter observational evaluation was conducted across 21 Italian rheumatology centers affiliated with the CReI group (Italian College of Rheumatologists), reflecting routine clinical practice in tertiary and secondary care settings throughout Italy. The aim of the evaluation was to assess patient characteristics and comorbidity profiles before and after the issuance of the PRAC recommendations concerning the use of JAK inhibitors.

Adult patients (≥18 years) with a confirmed diagnosis of RA, according to internationally accepted classification criteria, were eligible for inclusion if they had initiated or were receiving treatment with either JAK inhibitors or b-DMARDs. The b-DMARDs considered included tumor necrosis factor inhibitors (TNFi), interleukin-6 receptor inhibitors (IL-6Ri), abatacept, rituximab, and anakinra. No restrictions were applied regarding disease duration, line of therapy, or prior treatment exposure, in order to capture a real-world patient population.

The study population was divided into two distinct cohorts based on the timing of patient evaluation relative to the PRAC recommendations. The first cohort included 4421 patients evaluated before the release of the PRAC recommendations, between 1 January 2022 and 1 January 2023. The second cohort comprised 4376 patients evaluated after the implementation of the PRAC recommendations, between 2 January 2023 and 1 January 2024. For both cohorts, data were retrospectively extracted from medical records of patients attending routine outpatient visits at the participating rheumatology centers.

Demographic and clinical data were collected using standardized data extraction procedures at each center. In a preliminary analysis, the presence of major comorbidities known to be associated with increased cardiovascular and thromboembolic risk was assessed. These comorbidities included arterial hypertension, hypercholesterolemia, diabetes mellitus, prior myocardial infarction, history of deep vein thrombosis, and pulmonary embolism. Comorbidities were considered present if documented in the medical record or if the patient was receiving specific treatment for the condition.

In addition, lifestyle and risk-related variables were evaluated, including smoking status (current smoker, former smoker, or never smoker) and physician-assessed cardiovascular risk, as reported in the clinical records. Concomitant therapies relevant to cardiovascular and metabolic risk management were also recorded, including the use of antihypertensive agents, statins, acetylsalicylic acid, and oral antidiabetic medications. These treatments were used as indirect indicators of underlying comorbid conditions and overall risk profile.

Data were analyzed descriptively to compare baseline characteristics and comorbidity burden between patients evaluated before and after the PRAC recommendations. The primary objective of this preliminary analysis was to identify potential differences in patient selection, cardiovascular risk factors, and comorbidity profiles in the two time periods, reflecting changes in clinical practice following regulatory guidance. No intervention or modification of standard clinical care was performed as part of this evaluation.

### Statistical Analysis

Statistical analyses were performed to describe and compare demographic and clinical characteristics of the study population before and after the issuance of the PRAC recommendations. Descriptive statistics were used to summarize the data: categorical variables were reported as absolute frequencies and percentages (%), while continuous variables were expressed as mean values and standard deviations (SD), given the approximately normal distribution of the data.

The normality of continuous variables was assessed using standard distributional assumptions based on sample size and data dispersion. Comparisons between categorical variables, including the incidence of comorbidities and concomitant therapies, were conducted using Fisher’s exact test, which was chosen due to its robustness in handling differences in group sizes and low-frequency events. Comparisons between continuous variables were performed using the independent samples *t*-test.

To explore potential associations between clinical variables and outcomes of interest, multiple regression analyses were performed. In these models, one dependent variable was evaluated in relation to multiple independent variables, allowing adjustment for potential confounders. The selection of independent variables was based on clinical relevance and availability in the dataset. Results of regression analyses were expressed using appropriate effect estimates with corresponding confidence intervals, where applicable.

All statistical tests were two-sided, and a *p*-value of less than 0.05 was considered statistically significant. Statistical analyses were conducted using MedCalc Statistical Software, version 2023 (MedCalc Software Ltd., Ostend, Belgium).

## 3. Results

Table 2 provides a comprehensive overview of the demographic characteristics, cardiovascular risk profile, and comorbidity burden of patients with RA evaluated before and after the issuance of the PRAC recommendations. Overall, the two cohorts were largely comparable. A statistically significant reduction in the prevalence of myocardial infarction was observed in the post-PRAC cohort compared with the pre-PRAC cohort (0.47% vs 0.90%, respectively; *p* = 0.02). This finding represents the only major difference detected among the analyzed cardiovascular events. Mean age, sex distribution, smoking status, and the prevalence of other major comorbid conditions—including hypertension, hypercholesterolemia, diabetes mellitus, deep vein thrombosis, and pulmonary embolism—did not differ significantly between the two study periods, supporting the homogeneity of the populations analyzed and reducing the likelihood of selection bias related to baseline characteristics.

Beyond patient characteristics, the implementation of the PRAC recommendations was associated with substantial changes in therapeutic prescribing patterns in routine clinical practice. Overall, a redistribution of treatment choices across biologic and targeted synthetic DMARDs was observed. Prescriptions for monoclonal tumor necrosis factor inhibitors increased by 3.9% in the post-PRAC period, while the use of etanercept decreased by 2.6%, indicating a shift within the TNF inhibitor class itself. In parallel, prescriptions for IL-6 receptor inhibitors increased markedly, rising from 10.7% before the PRAC recommendations to 17% afterward. This change represents one of the most pronounced variations observed across all therapeutic classes and suggests an increased preference for IL-6 pathway inhibition following the regulatory guidance.

In contrast, the use of other biologic agents, including anakinra and abatacept, remained relatively stable across the two periods, with no statistically significant variations in prescription rates. This stability indicates that prescribing habits for these agents were not substantially influenced by the PRAC recommendations and may reflect their established safety profiles or more restricted indications in clinical practice.

As anticipated, JAK inhibitors were the therapeutic class most directly affected by the PRAC recommendations. A significant overall reduction in JAK inhibitor use was observed after the PRAC decision, with a cumulative decrease of 4.9% (*p* = 0.0003). However, the magnitude of this reduction varied considerably among individual JAK inhibitors. Baricitinib showed the largest decline in prescriptions (−3.0%, *p* = 0.0002), followed by filgotinib (−1.5%, *p* = 0.012) and tofacitinib (−1.4%, *p* = 0.0051). Notably, upadacitinib was the only agent within this class that was not significantly affected by the PRAC recommendations, displaying only a minimal and non-significant reduction of 0.3%. These findings indicate a heterogeneous impact of regulatory guidance within the JAK inhibitor class, as detailed in Table 3.

Despite these changes in prescribing behavior, the overall cardiovascular risk profile of the population, as assessed using validated risk stratification tools, remained unchanged. Cardiovascular risk evaluation performed using the ESC-SCORE and the Italian “Progetto Cuore” algorithms did not show significant differences between the pre- and post-PRAC periods, suggesting that the observed modifications in treatment patterns were not accompanied by a measurable change in estimated baseline cardiovascular risk [15,16]. Nevertheless, analysis of concomitant cardiovascular therapies revealed a significant increase in statin use following the PRAC recommendations, rising from 8.25% in the pre-PRAC period to 11.1% in the post-PRAC period (*p* = 0.05), as reported in Table 4. This finding may reflect increased attention to cardiovascular risk management in patients with RA during the post-PRAC period.

To understand the impact of these numerous variables, we performed a logistic regression, considering myocardial infarction as the dependent variable and the other items as independent variables. As can be seen in the table, only hypercholesterolemia and hypertension influence the risk of myocardial infarction.

Anyway, given myocardial infarction rates below 1%, multivariable modeling is likely underpowered, and cardiovascular findings should be interpreted descriptively (Table 5). Taken together, these analyses suggest that the observed reduction in myocardial infarction prevalence in the post-PRAC period cannot be attributed to differences in baseline cardiovascular risk factors or to exposure to specific DMARD therapies.

## 4. Discussion

This evaluation data indicate that PRAC recommendations have influenced the prescribing patterns of the JAKi ts-DMARD; moreover, a statistically significant reduction in myocardial infarction was observed in our population. Concurrently, a significant increase in the use of co-treatments, specifically statin therapy, was noted. Overall, these data corroborate findings from other existing registries and retrospective studies.

Data from the German RHADAR rheumatology registry demonstrated a significant reduction in the initiation of JAK inhibitor therapies following the PRAC recommendations, with JAKis increasingly prescribed as third-line or later treatment options in subsequent periods [17]. These findings align with evidence from a U.S. prescription-based study in RA, which reported a marked decline in JAKi use after the release of preliminary ORAL Surveillance results in January 2021, accompanied by a corresponding increase in prescriptions of tumor necrosis factor inhibitors (TNFi) [18]. More recently, a further retrospective analysis of the RHADAR registry revealed a higher treatment retention rate for JAKis compared with biologic DMARDs. Specifically, the five-year drug survival was greatest for JAKis (68.3%), followed by TNFi and interleukin-6 inhibitors (both 58.6%), abatacept (55.0%), and rituximab (53.3%) [19].

Furthermore, a study comparing RA patients who initiated tofacitinib treatment before (*n* = 2111) with those who initiated tofacitinib treatment after (*n* = 1664) January 2021, observed a decrease in mean age (64.1 vs. 63.0 years) and in the percentage of patients with cardiovascular comorbidities (all *p* < 0.01). These changes were significantly different from those observed in patients who initiated treatment with anti-TNF or non-anti-TNF biologics. Among patients receiving advanced active therapy, the probability of discontinuation was higher for tofacitinib than for TNF (hazard ratio 1.18, 95% confidence interval 1.10–1.26, *p* < 0.001). The higher rate of discontinuation of tofacitinib treatment was more pronounced in the presence of cardiovascular comorbidities (*p* < 0.05) [20]. A study of 232 patients (155 with baricitinib and 77 with tofacitinib) evaluated baseline patient characteristics regarding VTE (venous thromboembolism) risk factors. These were not statistically different when the JAKi was initiated before versus after the EMA warnings, although a trend toward a lower rate of VTE history was observed. Five VTE events occurred, four with baricitinib and one with tofacitinib. The study did not show a significant change in the characteristics of patients who initiated a JAKi after the EMA warnings. However, the lower rate of VTE history in patients after May 2019 suggests that rheumatologists considered the potential risk of VTE [21].

In our survey, we observed a significant reduction in myocardial infarction after the PRAC decision. Rheumatologists have likely learned to better manage traditional cardiovascular risk factors and have begun directly treating hypercholesterolemia, hypertension, and diabetes. Furthermore, the application of cardiovascular risk assessments has allowed rheumatologists to better assess, over a 10-year timeframe, which patients are at low risk and should be treated with JAKis [22,23]. A retrospective cohort study based on the Merative™ MarketScan^®^ Research Database, Merative L.P., Ann Arbor, MI, USA (2012–2021) evaluated adults aged 18–64 years with RA who initiated treatment with biologic or targeted synthetic DMARDs (b/tsDMARDs). Cox proportional hazards models were applied to estimate hazard ratios (HRs) and 95% confidence intervals (CIs) for the occurrence of major adverse cardiovascular events (MACE) within two years of treatment initiation, with adjustment for relevant confounding factors. Overall, 34,375 treatment exposures were analyzed, comprising 71% TNF inhibitors (TNFi), 10% JAK inhibitors (JAKi), 8% abatacept, 5% rituximab, and 5% interleukin-6 inhibitors (IL-6i). Rituximab was associated with the highest incidence of MACE (196 per 10,000 person-years; 95% CI 126–291), followed by IL-6i (111 per 10,000 person-years; 95% CI 57–193). Multivariable analyses indicated a numerically increased, though not statistically significant, risk of MACE with rituximab (HR 1.5; 95% CI 0.9–2.4) and IL-6i (HR 1.3; 95% CI 0.7–2.4), whereas no increased cardiovascular risk was observed with JAKi compared with TNFi use [24].

In addition, a meta-analysis of 42 studies with low-to-moderate risk of bias, encompassing 813,881 patients, reported a median age of 55.7 years (IQR 53.0–59.0) among JAKi users and 51.5 years (IQR 42.7–57.4) among TNF antagonist users, with women representing 76.5% of the study population. No significant difference in the incidence of serious adverse events was observed between patients treated with JAK inhibitors and those receiving TNF antagonists, with incidence rates of 0.72 (95% CI 0.56–0.92) and 0.66 (95% CI 0.49–0.89) per 100 person-years, respectively [25]. The survey revealed increased statin use after the PRAC recommendations, which may influence the reduction in myocardial infarctions. A recent post hoc analysis of the ORAL Surveillance trial evaluated patients with RA aged ≥50 years who had at least one additional cardiovascular (CV) risk factor and were treated with tofacitinib 5 mg twice daily (*n* = 1455), tofacitinib 10 mg twice daily (*n* = 1456), or a TNF inhibitor (TNFi; *n* = 1451). Statin use was assessed at baseline and throughout the study period. Among participants with established atherosclerotic cardiovascular disease (ASCVD) or high CV risk, 53.0% and 26.9%, respectively, were receiving statin therapy at baseline. Baseline statin utilization was comparable between the tofacitinib and TNFi treatment groups. Regardless of statin use, patients receiving tofacitinib exhibited greater increases in low-density lipoprotein (LDL) and high-density lipoprotein (HDL) cholesterol levels from baseline compared with those treated with TNFi. Among participants with a history of ASCVD who did not receive statins at any time during the study, the incidence of major adverse cardiovascular events (MACEs) was significantly higher in the tofacitinib group than in the TNFi group (HR 4.07; 95% CI 1.20–13.82). In contrast, among participants with prior ASCVD who received statin therapy either at baseline or during follow-up, no significant difference in MACE incidence was observed between tofacitinib and TNFi (HR 1.17; 95% CI 0.46–3.00) [26].

The decline in prescriptions affected all JAK inhibitors; however, the extent of the reduction varied among individual agents. Prescription rates for upadacitinib remained essentially stable, a fact supported by an extensive clinical development program; indeed, results from the SE-LECT-COMPARE study reported no safety signals related to major adverse cardiovascular events (MACE), deep vein thrombosis, or malignancies in RA patients treated with upadacitinib versus adalimumab [27]. 

A systematic review of 19 randomized controlled trials (RCTs), including 10,656 patients with a mean age of 51 years and follow-up durations ranging from 12 to 52 weeks, demonstrated increases in low-density lipoprotein cholesterol (LDL-C) and high-density lipoprotein cholesterol (HDL-C) following upadacitinib administration (3–48 mg/day) in 15 studies, while the LDL-C:HDL-C ratio remained unchanged. Pooled analyses of three placebo-controlled RCTs (*n* = 2577) showed that upadacitinib 15 mg was associated with increases in LDL-C of 15.18 mg/dL (95% CI 7.77–22.59) and HDL-C of 7.89 mg/dL (95% CI 7.08–8.69). Furthermore, a pooled analysis of 15 placebo-controlled RCTs (*n* = 7695) found no increased risk of MACE with upadacitinib (risk ratio [RR] 0.62; 95% CI 0.24–1.60) [28].

In an Italian cohort study including 194 patients, 57.9% were classified as ineligible for JAK inhibitor therapy according to the European Medicines Agency (EMA) ORAL Surveillance criteria. The most frequent reason for ineligibility was elevated MACE risk (70.2%), followed by age > 65 years (34.2%), smoking status (30.7%), increased risk of venous thromboembolism (VTE; 20.2%), and malignancy (7.0%). Application of the ERS-RA score [29] reduced the proportion of patients classified as having high cardiovascular risk to 18.6% (*p* < 0.001 compared with ORAL Surveillance criteria), thereby lowering the overall ineligibility rate to 46.4% [30].

The recent JAK-SWAP RER study evaluated patients with RA in remission who did not meet Italian Medicines Agency (AIFA) reimbursement criteria and either discontinued JAK inhibitors or switched to bDMARDs. After six months, remission or low disease activity was significantly less frequent among patients who modified their baseline therapy compared with those who continued JAK inhibitor treatment (76.7% vs 92.3%; *p* = 0.024) [31]. The PRAC recommendations have raised awareness of CVD risk assessment in the rheumatology community. A multidisciplinary steering committee composed of 11 members, including rheumatologists, a cardiologist, a hematologist with expertise in thrombophilia, and other researchers, conducted systematic searches of the literature, and the evidence was classified according to standard guidelines. Three general principles were defined. First, patients with chronic inflammatory rheumatic diseases have a higher risk of MACE and VTE than the general population. Second, rheumatologist has a central role in assessing the risk of cardiovascular disease (CVD) and VTE in patients with chronic inflammatory rheumatic diseases. Third, the risk of MACE and VTE should be regularly assessed in patients with chronic inflammatory rheumatic diseases, particularly before initiating targeted therapies. Eleven recommendations have been defined to prevent life-threatening complications of cardiovascular disease and TVE in patients with chronic inflammatory rheumatic diseases, providing a practical assessment of cardiovascular disease and TVE before considering the prescription of targeted therapies, and in particular JAKi [32]. Finally, the recommendations highlight the role of age as a limiting factor in prescribing JAK inhibitors. This data does not appear to impact the survey decisions, as the mean age of patients before and after the PRAC was 46.1 ± 23.3 years compared to 50.8 ± 21.9 years. A study in RA patients treated with filgotinib. After six months of treatment, the elderly population > 65 years of age showed higher LDL cholesterol levels and lower HDL cholesterol levels compared to younger patients < 65 years of age. The atherogenic index and coronary risk index were higher in patients ≥ 65 years of age, but interestingly, no differences were found when comparing the 6-month data with baseline values [33].

## 5. Conclusions

Despite the non-interventional and retrospective design of this survey, our findings indicate that PRAC recommendations significantly influenced JAK inhibitor prescribing patterns in Italy, fostering more cautious and risk-adapted therapeutic decision making in patients with RA. Notably, these measures promoted a more structured approach to cardiovascular risk assessment and management, rather than merely limiting access to JAK inhibitors.

Further real-world studies with longer follow-up are needed to adequately evaluate rare but clinically relevant outcomes, including malignancies and thromboembolic events, and to better define the long-term cardiovascular safety of JAK inhibitors in RA patients with cardiovascular risk factors. However, current real-world evidence and long-term extension data from pivotal clinical trials suggest no increased cardiovascular risk when JAK inhibitors are appropriately prescribed. This survey represents an initial step in assessing the real-world impact of regulatory guidance on RA treatment strategies. In line with personalized medicine principles, future research should integrate clinical features, comorbidities, molecular and biomarker data, and advanced analytical tools, including artificial intelligence-based models, to refine patient stratification and optimize treatment selection. As members of the CReI network, we advocate for continued multicenter investigations across other immune-mediated rheumatic diseases, such as spondyloarthropathies and psoriatic arthritis, to support safer and more individualized care.

## Figures and Tables

**Table 1:** Pharmacovigilance Risk Assessment Committee (PRAC) recommendations for the use of JAK inhibitors.

- Age over 65
- Smoking
- History of MACE, VTE, and cancer
- Serious infections and all-cause mortality

**Table 2:** Population characteristics and comorbidities before and after PRAC recommendations.

	Before PRAC	After PRAC	*p*
Number Patients	4421	4376	
Average Patients Center	210.5 ± 177.4	208.3 ± 183.7	NS
Age	46.1 ± 23.3	50.8 ± 21.9	NS
Female/Male	79.5/20.5%	78.8/21.2%	NS
Smokers	8.60%	8.40%	NS
Hypertension	8.70%	9.70%	NS
Hypercholesterolemia	8.30%	7.20%	NS
Diabetes	4.50%	5.20%	NS
Myocardial Infarction	0.90%	0.47%	0.02
Deep Vein Thrombosis	0.40%	0.52%	NS
Pulmonary Embolism	0.20%	0.30%	NS
1-line treatment	117	96	NS
2-line treatment	1113	1133	NS
3-line treatment	2806	2814	NS
4-line treatment	385	333	NS

NS: not significant.

**Table 3:** Percentage of b-DMARDs and tS-DMARDs before and after PRAC recommendations.

% Average Treated Patients	Before PRAC	After PRAC	*p*	% Differences
Anti-TNFα	43.7	47.6	0.025	3.9
Etanercept	17.3	14.7	0.004	−2.6
Anti IL-6	10.7	17	0.0001	6.3
Anti IL-1	2.5	2	NS	−0.5
Abatacept	8.3	7.7	NS	−0.6
Rituximab	3	4.5	0.0001	1.5
JAK Inibitori (total)	22.2	17.3	0.0003	−4.9
Tofacitinib	6	4.6	0.0051	−1.4
Baricitinib	10	7	0.0002	−3
Upadacitinib	7.4	7.1	NS	−0.3
Filgotinib	8.4	6.9	0.012	−1.5

NS: not significant.

**Table 4:** CV risk score and co-treatments.

	Before PRAC	After PRAC	*p*
Antihypertensives	8.60%	8.30%	NS
Acetylsalicylic acid	0.85%	0.83%	NS
Statins	8.25%	11.1%	0.05
Oral antidiabetics	4.45%	5.20	NS
Both SCORE	23.8%	23.8%	NS
ESC-SCORE	23.8%	23.6%	NS
Progetto-Cuore	4.7%	4.9%	NS

NS: not significant.

**Table 5:** Logistic regression.

Coefficients and Standard Errors						
Variable	Coefficient	Std. Error	Wald	*p*	Odds Ratio	95% CI
Abatacept	0.78287	0.52886	2.1913	0.1388	2.1877	0.7959 to 6.1684
Anti IL1	0.62596	0.41446	2.281	0.131	1.87	0.83 to 4.2135
Anti IL6	−0.67771	0.36519	3.4439	0.0635	0.5078	0.2482 to 1.0388
Anti TNFalfa	0.018	0.07321	0.061	0.8049	1.0183	0.8821 to 1.1754
Etanercept	0.0997	0.08169	1.4898	0.2223	1.1049	0.9414 to 1.2967
Rituximab	−0.38308	0.29474	1.6893	0.1937	0.6818	0.3826 to 1.2148
JAK-tot	0.18051	0.10689	2.8517	0.0923	1.1978	0.9714 to 1.4770
Baricitinib	−0.65758	0.37509	3.0734	0.0796	0.5181	0.2484 to 1.0807
Tofacitinib	0.02863	0.30051	1.9278	0.9141	1.029	0.5710 to 1.8545
Filgotinib	0.17886	0.09491	3.551	0.0595	1.1959	0.9929 to 1.4404
Upadacitinib	−0.10155	0.19464	2.388	0.1223	0.7777	0.5654 to 1.0698
Hypercolesterolemia	0.65868	0.27715	5.6482	0.0175	1.9322	1.1224 to 3.3264
Hypertension	−0.66805	0.2679	6.2184	0.0126	1.5127	1.3033 to 1.8668
Diabetes	−0.11027	0.23282	0.2243	0.6358	0.8956	0.5675 to 1.4135
Age	−0.12856	0.068024	3.5717	0.0588	0.8794	0.7696 to 1.0048

## Data Availability

The original contributions presented in this study are included in the article. Further inquiries can be directed to the corresponding author.
